# A Rare Case of Aggressive Digital Adenocarcinoma of the Lower Extremity, Masquerading as an Ulcerative Lesion that Clinically Favored Benignancy

**DOI:** 10.3390/healthcare2030315

**Published:** 2014-08-27

**Authors:** Ryan Vazales, Dustin Constant, Robert J. Snyder

**Affiliations:** 1 Department of Podiatric Medicine and Surgery, Florida Hospital East Orlando, Lake Underhill Road, Orlando, FL 32822, USA; 2 Barry University School of Podiatric Medicine, Miami Shores, FL 33161, USA; E-Mail: dustin.constant@mymail.barry.edu; 3 Clinical Research and Fellowship Program, Barry University School of Podiatric Medicine, Miami Shores, FL 33161, USA; E-Mail: drwound@icloud.com

**Keywords:** papillary, adenocarcinoma, adnexal carcinoma, metastatic ulceration, cutaneous carcinoma

## Abstract

A rare case report of Aggressive Digital Adenocarcinoma (ADPCa) is presented complete with a literature review encompassing lesions that pose potential diagnostic challenges. Similarities between basal cell carcinoma (BCC), marjolin’s ulceration/squamous cell carcinoma (MSCC) and ADPCa are discussed. This article discusses potential treatment options for ADPCa and the need for early biopsy when faced with any challenging lesion. An algorithmic approach to ADPCa treatment based on the most current research is recommended.

## 1. Case Report

An 85 year-old black female with a 7 year history of type II diabetes mellitus presented with a chief complaint of long standing repeated foot infections to the left big toe. The wound on the left hallux was consistent with a typical recalcitrant foot ulceration in a patient with diabetes mellitus (DFU). The patient stated that in the past, the wound healed with antibiotic and wound care treatment over a 6–10 week period and then often would reopen over the same time frame. According to the patient’s daughter, the current ulceration has been open for a two year period despite continued attempts at healing including advanced treatment of surgical debridement with placement of a skin graft. These modalities were met with little success in getting the wound to close.

Much of the patient history was gathered via communication with the patient’s daughter, who stated that the ulceration to the left big toe had been “occurring on and off for 20 years”. The patient’s past medical history included diabetes mellitus type II of seven years duration, chronic venous insufficiency; arthritis of both knees; hypertension of 15 year duration and high cholesterol for 2 years. The patient stated that she felt her foot problems began at age 21. Surgical history included hernia repair 11 years ago and hysterectomy 9 years ago. A review of the patient’s family history revealed DM type II; heart disease; high blood pressure; arthritis and colon cancer for the patient’s mother; medical history was unknown for her father; colon cancer for her brother and heart disease and high blood pressure were noted for her sister. The patient’s medication list was not available at the time of her consultation. The patient’s allergies included reaction to “ace inhibitor medication,” which caused her to cough. The patient denied smoking or the use of alcohol or recreational drugs.

## 2. Physical Exam

**Figure 1:**
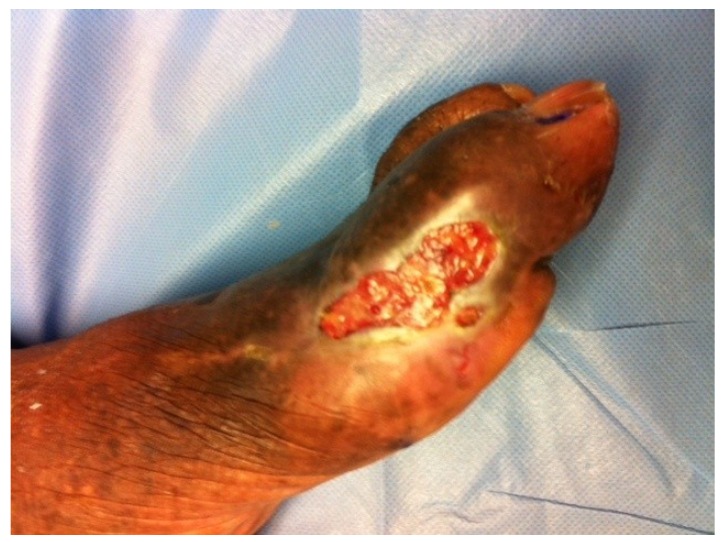
Aggressive digital adenocarcinoma presenting as a typical recalcitrant (DFU).

Physical examination revealed an ulcer over the area of the medial and dorsal aspect of the left hallux that measured 3cm circumferential area with no probing to deeper layers and no undermining present. The skin over the area was dry with no exudates, satellite lesions or periwound erythema noted. No secondary signs of infection including edema, foul odor or pain out of proportion were noted. The wound base was composed of a 50/50 mix of fibrous and granulation tissue (Figure 1). Additionally, varicosities, lipo-dermatosclerosis and hemosiderin deposits were also present, however no edema was noted bilaterally to the distal lower extremity. Skin temperature from proximal to distal lower extremity was warm to warm, and pedal pulses were present but diminished bilater­ally. Doppler exam revealed biphasic wave forms to both lower extremities and an ABI of the left lower extremity showed a ratio of 0.994. Capillary refill time was within normal limits to bilateral lower extremities. Neurologic exam revealed vibratory and sharp/dull sensations to be completely absent to the bilateral lower extremity. Furthermore, protective threshold was absent per a Semmes Weinstein 5.07 monofilament test in 10 locations bilaterally. Deep tendon reflexes were within normal limits bilaterally. Active and passive range of motion of the ankle joints and pedal joints produced no limitation, crepitus, or pain, and muscle strength was within normal limits bilaterally. X-ray exams were noncontributory showing uniform bone density and no signs of cortical erosion or clinical osteomyelitis.

With no history of previous cancer, there was little evidence based on appearance alone, to suggest the possibility of a tumor. However, due to the length of time having the recalcitrant wound, the lesion raised concern over the possibility of tumor formation. This provided a possible differential diagnosis that included amelanotic melanoma (AM), carcinoma cuniculatum (CC), basal cell carcinoma (BCC) and marjolins squamous cell carcinoma (MSCC). All of these diagnoses are capable of presenting as ulcerative lesions and often indicate early biopsy in multiple locations and re-biopsy as part of the treatment plan. Furthermore, BCC and MSCC usually grow over a long period of time, similar to that of ADPCa.

## 3. Diagnosis

A standard blood panel for chemistry and hematology was taken but values were found to be not clinically significant. Three millimeter punch biopsies were taken at the wound sight in multiple locations. Using the clock face technique for distinguishing punch locations, biopsies were taken at 12:00 (proximal) and 06:00 (distal). These biopsies were sent in formalin medium to pathology services for further evaluation in hopes of finding a definitive diagnosis.

The pathology report indicated features of eccrine differentiation with atypical epithelial cells consistent with low grade adnexal proliferation. Lesional cells exhibited extension into the superficial and deep peripheral surfaces of the specimen. Although the proliferation was assymetrical, nuclear atypia was not severe suggesting a low grade sweat duct tumor which favored benignancy; however, malignancy could not be ruled out. Given the differential diagnosis stated above, poor outcomes with standard wound care to date and no clear disease process identified, a second set of biopsies was indicated. After follow up conversations with the pathologist, as well as patient and family, a second set of biopsies were taken using the same technique at 3 and 9 o’clock, as well as, the central base of the lesion. Again, they were sent to pathology for evaluation. The pathology report for the second set of biopsies showed findings of infiltrative neoplastic cells with moderate cytologic atypia in all three locations consistent with Aggressive digital papillary adenocarcinoma (intermediate to high grade) also known as ADPCa, a much more serious disease process.

## 4. Treatment

Treatment rendered while waiting for pathology results included sharp debridement of nonviable soft tissue and the use of cadexomer iodine applied to the wound base along with a dry sterile dressing. The patient was instructed to continue with dressing changes every other day to keep the wound clean, dry and free of bacterial pathogens and to return to clinic in 1 week for further evaluation and review of pathology results. Once a clear diagnosis was made based on pathology, both conservative and surgical treatment options were explained and consults for oncology, vascular surgery, plastic surgery, as well as, palliative care management was highly advised. The patient was referred to oncology. Follow up with oncology revealed that based on the patient’s age and physicality, she and her family elected to treat conservatively with palliative care.

## 5. Discussion

This case presents a potential diagnostic challenge. The possibility of tumors/cancers masquerading as wounds can be serious and treatment plans require a clinician’s keen eye regarding clinical picture and patient history. The clinician’s discretion to biopsy early can potentially minimize devastating long term results and maximize best treatment practices. Thus, a literature review was undertaken looking at both ADPCa, as well as, some other cancer/disease processes that follow similar growth patterns, present with similar clinical characteristics and have a potential to metastasize.

### 5.1. Basal Cell Carcinoma

BCC is the most common skin tumor reported in the literature worldwide and arises from the epidermis. Although it is a generally slow growing, often painless tumor, BCC has the potential and rare occasion to metastasize. Due to its slow growing nature, basal cell tumors allow for a successful treatment outcome with surgical excision in 90% of the cases [1,2]. Its name is derived from the cells that resemble the basal layer of the epidermis often described as “Basaloid”. The etiology of BCC is thought to be multifactorial with risk factors including UV light exposure, tanning beds, ionizing radiation and recipients of solid organ transplants [3]. Although both the P53 tumor suppressor mutation and the Patched (PTCH) gene have been well documented to be associated with BCC, an article by Nghiem *et al.* 2003 [4] revealed the potential of UVA radiation to act as a tumor promoter by activating protein Kinase C, as well as, a cutaneous immunosuppressor ultimately leading to malignant transformation. Even more was the discovery that the combination of different wavelengths, *i.e.*, UVB in conjunction with UVA, work synergistically to activate the malignant transformation in cutaneous cells [4].

The literature has provided some similarities of BCC to ADPCa. One such similarity is BCC’s ability to present with a number of different clinical scenarios, from a nodular lesion to a tissue destructive ulceration. In fact, most primary metastatic BCC present as destructive tissue lesions representing up to 75% [5]. Another similarity of BCC to ADPCa is the high risk of recurrence after surgical resection of the lesion. A meta-analysis of the data by Marcil and stern in 2000 [6] revealed that after a review of 9005 patients, the risk of recurrence with BCC after resection was 44% within a 3 year period. Tefler* et al*. in 2008 [7] described risk factors affecting the level of recurrence and prognostic outcome with BCC to include: tumor size, site, margins, histological subtype and aggression, failure of previous treatment and immunosuppression [7]. Subtypes of BCC include superficial, morpheaform, fibroepithelioma of pinkus, pigmented and common. Furthermore, both ADPCa and BCC have multiple treatment options available. However, surgical excision appears to be the standard of care due to the concern of malignant transformation. For this reason, early biopsy should be sought in wounds that present with BCC or ADPCa characteristics and thorough follow up is indicated, especially in recalcitrant or challenging lesion presentations.

### 5.2. Marjolin’s Ulceration/Squamous Cell Carcinoma (MSCC)

Traditionally, a Marjolin’s ulcer is a rare malignancy that arises from a chronic nonhealing ulcer. Although most commonly associated with burns, it has been shown that MSCC can be in concomitances with many other pathologic processes. These include but are not limited to; pressure ulcers, chronic venous ulcers, osteomyelitis, traumatic wounds, fistulas, and lacerations [8]. While much of the etiology is still unclear, there is a consensus that MSCC stems from a multifactorial mechanism that contributes to the malignancy. It was originally hypothesized that scar tissue has impaired immunological response due to the lymphatic damage and decreased vascularity. This compromised immune response then allows for further uncontrollable mutations, which lead to the malignancy [9]. More recently, it has been suggested that predisposed immune deficient individuals along with chronic irritation may play a significant role. Chronic irritation causes continuous mitotic turnover, along with avascularity and depressed immune response allowing for malignant cell proliferation [10]. Histologically, majolin’s ulcer present as MSCC and BCC in 83% present of the cases. The majority (73%) being that of MSCC, is important due to its high potential for metastasis [11]. Metastasis rates of MSCC approach upwards of 54% of the cases and presentation in the lower extremity in 50% of the cases [12].

Due to its clinical presentation in the form of an ulcerative lesion and it’s potential to metastasize, MSCC must also be considered in the differential diagnosis for ADPCa. MSCC often presents 25 years after the initial cutaneous damage. This significant latency period is similar to that of ADPCa [8]. The suggestion of diagnosis for MSCC follows a distinct triad of nodular formation, induration, and ulceration which are all present in BCC and ADPCa [10]. As with BCC and ADPCa, recurrence rates can approach 50% with inadequate resection [13]. Furthermore, many of the treatment options to be considered for MSCC are similar to that of BCC and ADPCa. Treatment includes wide local excision of at least 1 centimeter healthy margin or amputation when margins cannot be confirmed.

There has been some discussion on the importance of performing frozen section examinations intra-operatively. While this seems to be an ideal technique, it is important to note that the quality of frozen sections are very low and might not be as predicable for lesions which have many morphologies [14]. Holgado and Ward in 2000 [14] suggested that the Mohs surgical technique might provide the best outcome for removing all cancerous tissue and salvaging as much viable tissue as possible. In theory, this would be the best option for the treatment of all malignant ulcerative lesions, however it is unrealistic considering that the physician would need to be trained in surgery and pathology.

### 5.3. Aggressive Digital Papillary Adenocarcinoma (ADPCa)

Aggressive digital papillary adenocarcinoma (ADPCa), first described by Kao and Helwig in 1984 [15], is a rare variant of sweat gland carcinoma. It is a metastatic cutaneous tumor that predominantly occurs on digits and volar surfaces of middle aged to older males [16,17]. Some risk factors include previous radiation therapy and immunosuppression [18]; however, the pathophysiological mechanism of this disease process is still largely unknown. In 2012, Suchak [19] revealed that of 31 cases reviewed, only 4 presented in the lower extremity indicating the even more rare presentation seen in this particular case. ADPCa differentiates itself from other sweat gland carcinomas by its potential capability to metastasize quickly and high recurrence rate. Even with surgical excision, recurrence rates of up to 42% have been reported [20]. Furthermore, after reviewing 19 cases of ADPCa, Hsu *et al*. in 2009 [16] showed that metastasis was described in 47% of the cases, and local reoccurrence was seen in 50% of the cases within 2 months of excision, indicating the difficulty in rendering successful treatment.

Diagnosis of ADPCa is also challenging as some authors have suggested a mixed adnexal lineage with eccrine, apocrine and pilosebaceous features [21]. Also, ADPCa can often present with common clinical characteristics to other lower extremity lesions. This allows these tumors to masquerade as benign lesions such as nodules or papules and even ulcerations as seen in this particular case [22,23]. Most often, the legion presents as a nodule or papule that is mildly painful and relatively slow growing [24] which adds to the difficulty in diagnosis when presented with an ulcerative lesion. Many times the clinical presentation can represent that of a ganglion cyst [22]. Histological characteristics of ADPCa include lobulated infiltrating neoplastic cells often with a focal papillary growth pattern. These neoplastic cells often show cytologic atypia with pleomorphism representing more than one morphologic subtype. Spindle cells with heavily active mitosis may also be seen. Even histology does not necessarily allow for a definitive diagnosis, as ganglion cysts, synovial sarcomas and fibrosarcomas all have histologic characteristics similar to ADPCa [22]. Most recently Suchak* et al*. in 2012 [19], stated that histological parameters currently used for diagnosis are not reliable in predicting the potential for metastasis. Thus all tumors with any remote characteristics of ADPCa should be treated as such until proven otherwise.

### 5.4. Proposed Treatment

Currently there are a number of plausible treatment recommendations for aggressive digital papillary adenocarcinoma. However, a guide to treatment has not been well defined in literature due to its rarity. When Kao and Helwig first introduced ADPCa in 1987 [20] the standard of care included local excision of the tumor. Research by Tsujita-Kyutiku *et al*. in 2003 [25] discussed the use of epithelial markers, specifically the P63 marker to help differentiate low grade from high grade ADPCa. Di Como *et al*. [26] showed that P63 staining would be positive in the basal layers of normal sweat ducts and primary carcinomas but negative in metastatic foci indicating that it may allow the ability to determine whether amputation or excision is warranted. However, the data continues to be inconclusive. The malignant nature of ADPCa has warranted more aggressive therapy such as a more extensive excisional debridement and even amputation of the affected digit or limb [16]. In fact, in the Hsu *et al*. 2009 [16] study, amputation was the method of treatment in 11 out of 19 cases. However, it should be noted that although amputation is recommended with recurrent ADPCa, no statistical significance regarding outcomes was found when compared to the other 7 of 18 case reports reviewed that underwent wide excision with/without sentinel node biopsy.

More recently, the approach towards APDCa has become much more targeted due to a new emphasis on limb preservation. A few case studies have suggested chemotherapy as a viable option for treatment of ADPCa although the outcomes where only marginally beneficial [22,27]. In a study by Jones* et al.* 2013 [17], the authors suggest conventional palliative doses of radiotherapy as an adjuvant to chemotherapy for the treatment of ADPCa. However, the role of radiation and chemotherapy in the treatment of ADPCa is still uncertain.

Sentinel lymph node biopsy combined with local excision has also been recommended to detect subclinical metastases [28]. While this may be a pivotal step in detecting early metastasis, it has been documented that patients can have a prolonged disease-free interval of up to 20 years after the initial presentation before seeing recurrence making this disease process a challenge for physicians.

## 6. Summary

While there has been some inconsistency within the literature on how to approach the treatment for difficult lesions such as BCC, MSCC and ADPCa, the similarities in clinical presentation indicate the need to biopsy early and many times in multiple locations and more than once for definitive diagnosis. Early biopsy in challenging and recalcitrant wounds can provide the best information to make an informed decision on treatment options. It is important to note that many of the treatment options discussed regarding ADPCa have only been reported in small case studies. The infrequent occurrence of ADPCa in the lower extremity presents a diagnostic dilemma often being diagnosed incorrectly. ADPCa is a rare cutaneous metastatic carcinoma and physicians should treat any lesions exhibiting characteristics consistent with the disease process as such.

Clinical examination and history still appear to be the best diagnostic tools at our disposal. As with any ulcerative presentation, we must take the time to examine the signs of malignancy which include chronic ulceration greater than 3 months, rolled or everted wound borders, boisterous granulation tissue, foul smelling purulence, increase in size, bleeding on contact, and pain. If any of these criteria are present, it is our obligation to consider biopsy for definitive diagnosis.

Our literature review revealed no clear guidelines for re-biopsy of suspicious lesions even though the author’s feel that this practice is indicated with recalcitrant wounds that remain problematic. Further research is needed to better elucidate a successful treatment guideline that can be followed based on clinical evidence, clinical picture, lab results and physician expertise. Perhaps most important is the clinicians’ discretion to biopsy and re-biopsy when faced with ambiguous results or recalcitrant wounds. Based on the most current literature, the authors suggest a Sentinel lymph node biopsy during the first wide excision, along with an annual exam for reoccurrence and chest x-ray to evaluate for lung metastasis. This, combined with repeat biopsies in multiple locations when indicated, may provide the best algorithmic approach and produce significantly better outcomes to these rare and challenging disease processes.
